# A rare case of dual left anterior descending artery type 4 in inferior myocardial infarction patient: a case report

**DOI:** 10.1093/ehjcr/ytaf032

**Published:** 2025-02-05

**Authors:** W Yus Haniff W Isa, Zurkurnai Yusof, Mohamad Fadini Abdul Aziz, Mohd Khairi Othman, Ayman Suliman

**Affiliations:** Cardiology Unit, Hospital Universiti Sains Malaysia, Kubang Kerian, 16150 Kelantan, Malaysia; Department of Internal Medicine, Hospital Universiti Sains Malaysia, Kubang Kerian, 16150 Kelantan, Malaysia; Cardiology Unit, Hospital Universiti Sains Malaysia, Kubang Kerian, 16150 Kelantan, Malaysia; Department of Internal Medicine, Hospital Universiti Sains Malaysia, Kubang Kerian, 16150 Kelantan, Malaysia; Department of Internal Medicine, Hospital Universiti Sains Malaysia, Kubang Kerian, 16150 Kelantan, Malaysia; Cardiology Unit, Hospital Universiti Sains Malaysia, Kubang Kerian, 16150 Kelantan, Malaysia; Department of Internal Medicine, Hospital Universiti Sains Malaysia, Kubang Kerian, 16150 Kelantan, Malaysia; Department of Internal Medicine, Shendi University, PO Box 142-143 Shendi, Sudan

**Keywords:** Myocardial infarction, Dual LAD, Percutaneous coronary intervention, Cardiac imaging, Case report

## Abstract

**Background:**

This case highlights a type 4 dual left anterior descending coronary artery anomaly, identified incidentally during coronary angiography.

**Case summary:**

A 51-year-old male patient presented with acute myocardial infarction, which was successfully treated with thrombolysis and percutaneous coronary intervention. Angiography revealed an anomalous origin of the left anterior descending artery (LAD) from the right coronary artery. Stenting was performed on the right coronary artery and the right posterior descending artery branch.

**Discussion:**

Type 4 dual LAD is a rare coronary anomaly with potential clinical risks, including sudden cardiac death due to its course between major arteries. Variations in the structure of coronary arteries can present challenges for interventional cardiologists when conducting percutaneous coronary interventions. Accurate coronary anatomy assessment via angiography and computed tomography coronary angiography is crucial for successful percutaneous coronary intervention and surgical planning. Although uncommon, the dual LAD type 4 anomaly is a significant coronary artery variation that interventional cardiologists must consider due to its impact on prognosis and long-term treatment strategies.

**Learning points**

- To increase awareness of the anatomical variants of dual LAD, which vary from type 1 to type 4 depending on where the short and long LAD originate and run.
- Dual LAD can be misinterpreted as a single LAD with severe stenosis or occlusion, leading to inappropriate treatment strategies. Therefore, understanding the exact anatomy is crucial for planning revascularization procedures.
- Utilize imaging such as CTCA/MRI cardiac to conduct a thorough assessment of both the short and long LAD segments to determine the extent of the coronary artery disease or abnormalities.

## Background

Congenital coronary anomalies are rare and reported to occur in 0.64%–1.3% of patients undergoing coronary angiography.^1^ Within this context, we present a rare case of type 4 dual left anterior descending artery (LAD), incidentally discovered during coronary angiography in a patient with ST-segment elevation myocardial infarction (STEMI). Normally, the left main coronary artery (LMCA) emerges from the left coronary sinus and gives rise to LAD, which courses towards the cardiac apex in the anterior interventricular sulcus (AIVS).^2^ Dual LAD is a term used to describe a class of anomalies where the LAD has two distinct ostial sources, or it could be divided into two primary divisions of LAD to supply the same territories.^3^ The incidence of dual LAD varies from 0.68% to 6% in different case series. The rarest and perhaps the most challenging variant is dual LAD type 4, which accounts for 0.004%.^4^ This can always inadvertently be diagnosed during coronary angiography. The first description and classification of dual LAD was made in 1983 by Spindola-Franco *et al*.^5^ He described 23 cases of dual LAD anomalies, classifying them into four subtypes: type 1, where the long LAD runs in the AIVS on the left ventricular side before re-entering the distal AIVS; type 2, where the long LAD descends on the right ventricular side before re-entering the AIVS; type 3, in which the long LAD courses intramyocardially within the ventricular septum; and type 4, where the long LAD originates from the right coronary artery (RCA), with the short LAD and proper LAD forming a very short vessel high in the AIVS, giving rise to major septal perforators and diagonal branches.

## Summary figure

**Figure:**
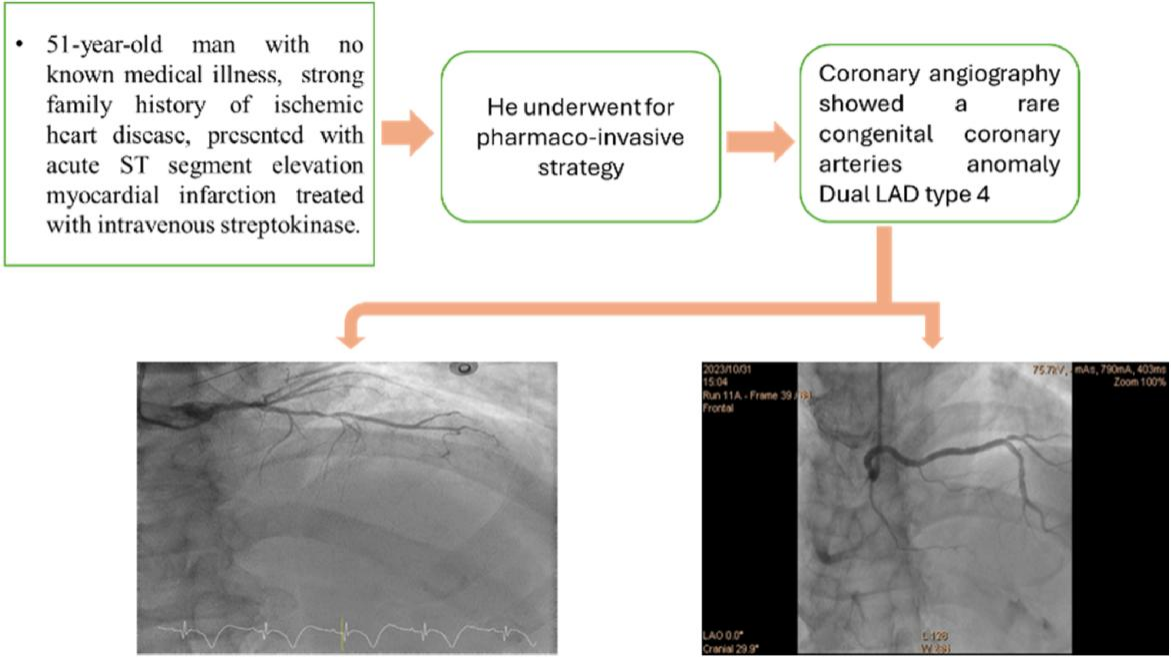


## Case presentation

**Figure 1:**
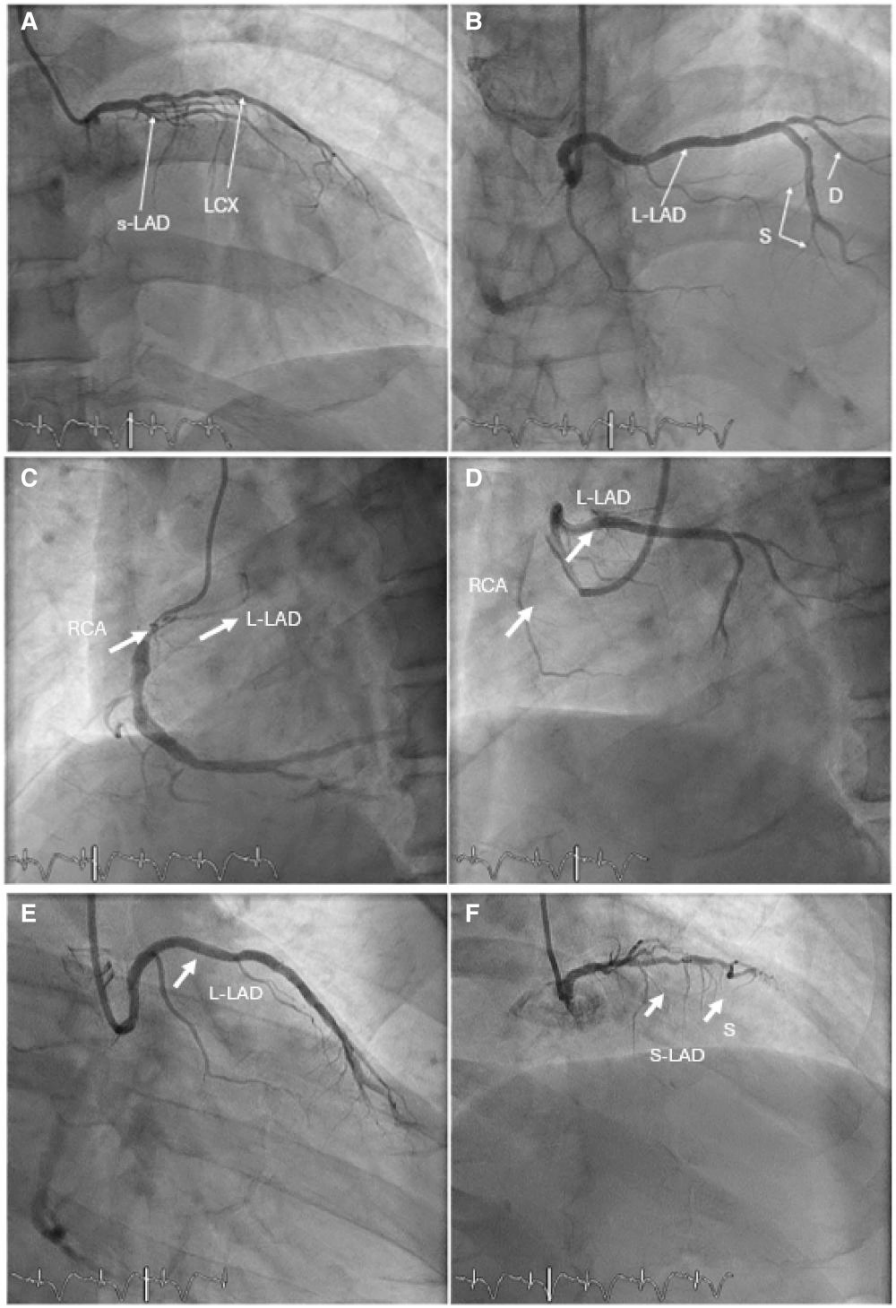
(*A*) Left anterior oblique cranial view shows a short left anterior descending artery with small septal branches and diagonal arising from the same. The left circumflex is normal. (*B*) Left anterior oblique cranial view showing a long left anterior descending artery is seen arising from the proximal right coronary artery, as it traverses across towards the anterior interventricular sulcus. A diagonal branch arising from long left anterior descending artery is also seen and septal branches. (*C*) Left anterior oblique caudal view showing a proximal right coronary artery lesion, located just beyond the origin of the long left anterior descending artery. (*D*) Left anterior oblique caudal view displaying a proximal right coronary artery lesion, located just beyond the origin of the extended left anterior descending artery. (*E*) Right anterior oblique caudal view showing long course of proximal part of long left anterior descending artery as it traverses across towards the anterior interventricular sulcus. A diagonal branch arising from long left anterior descending artery is also seen. (*F*) Right anterior oblique cranial shows a short left anterior descending artery with small septal branches and diagonal arising from the same. S-LAD: short Left anterior descending artery; L-LAD: long Left anterior descending artery; S: septal branches; D: diagonal branches; RCA: right coronary artery; LCX: left circumflex.

A 51-year-old man complained of sudden onset of central chest pain 1 h prior to his presentation to the hospital. It was heavy in nature, with a pain score of 8 out of 10, radiating to his neck. The pain began while he was at rest, with no identified worsening and alleviating factors. It was associated with nausea, profuse sweating, and dyspnoea. The patient did not report any previous episodes of similar pain. He denied experiencing palpitations, dizziness, or syncope. There was no history of recent fever, cough, palpitation, or upper respiratory symptoms. The patient has no history of recent travel, trauma, or prolonged immobility, and he has no known risk factors for venous thromboembolism. He was an active smoker of 30 pack-years and has history of kratom (*Mitragyna speciosa*) leaf abuse. He has a strong family history of coronary heart disease that involved his younger and elder brothers. Otherwise, there was no family history of sudden death, and he has no known medical illness. Upon arrival in the emergency department, he was alert and conscious, afebrile, with a pulse rate of 55 b.p.m., with a good volume, and regular rhythm. His blood pressure was 103/66. The respiratory rate was 20 breaths per minute, and his oxygen saturation was 100% on room air. On inspection, the jugular venous pressure was elevated. The apex beat was palpated in the fifth intercostal space at the midclavicular line and was not displaced. Auscultation revealed normal S1 and S2 heart sounds, with no audible murmurs. There was bilateral basal fine crepitation, and other systems were unremarkable. The blood investigations have showed a haemoglobin 14.5 g/dL, white blood cell 15 × 103/μL, platelet 361 × 103/μL, serum sodium 138 mmol, serum potassium 3.5 mmol, serum urea 4.6 mmol, serum creatinine 131 mmol, serum aspartate transaminase 20 mmol, serum alanine transaminase 18 mmol, and serum alkaline phosphatase 71 mmol. The electrocardiogram (ECG) has showed a sinus rhythm with ST-segment elevation over the inferior leads (II, III, and AVF) and the right-sided lead (V4R). The chest radiography has showed cardiomegaly with an enlarged heart silhouette. The transthoracic echocardiography has revealed a left ventricular ejection fraction of 46% with hypokinesia of the septal, inferior, and posterior wall. He was diagnosed with acute heart failure (Killip2 classification) due to acute ST-segment elevation inferior myocardial infarction with the right-sided involvement. He was given a crushed oral aspirin 300 mg, oral clopidogrel 300 mg, and an intravenous infusion of 1.5 megaunits of streptokinase, which was administered to the patient within 10 min of medical contact (as our facility is limited to performing elective percutaneous coronary intervention (PCI) and does not provide primary PCI services). Availability and cost limitations may have restricted the use of ticagrelor or prasugrel, leading to the selection of clopidogrel as a more practical alternative. Serial ECGs demonstrated a reduction in ST-segment elevation by >50% within 30 min and almost complete resolution within 90 min ([Supplementary material online, *Figure 1* will include serial ECGs, providing a chronological representation of the patient's electrocardiographic changes]). The thrombolysis procedure was uneventful, and it was followed by a successful reperfusion.

**Figure 2:**
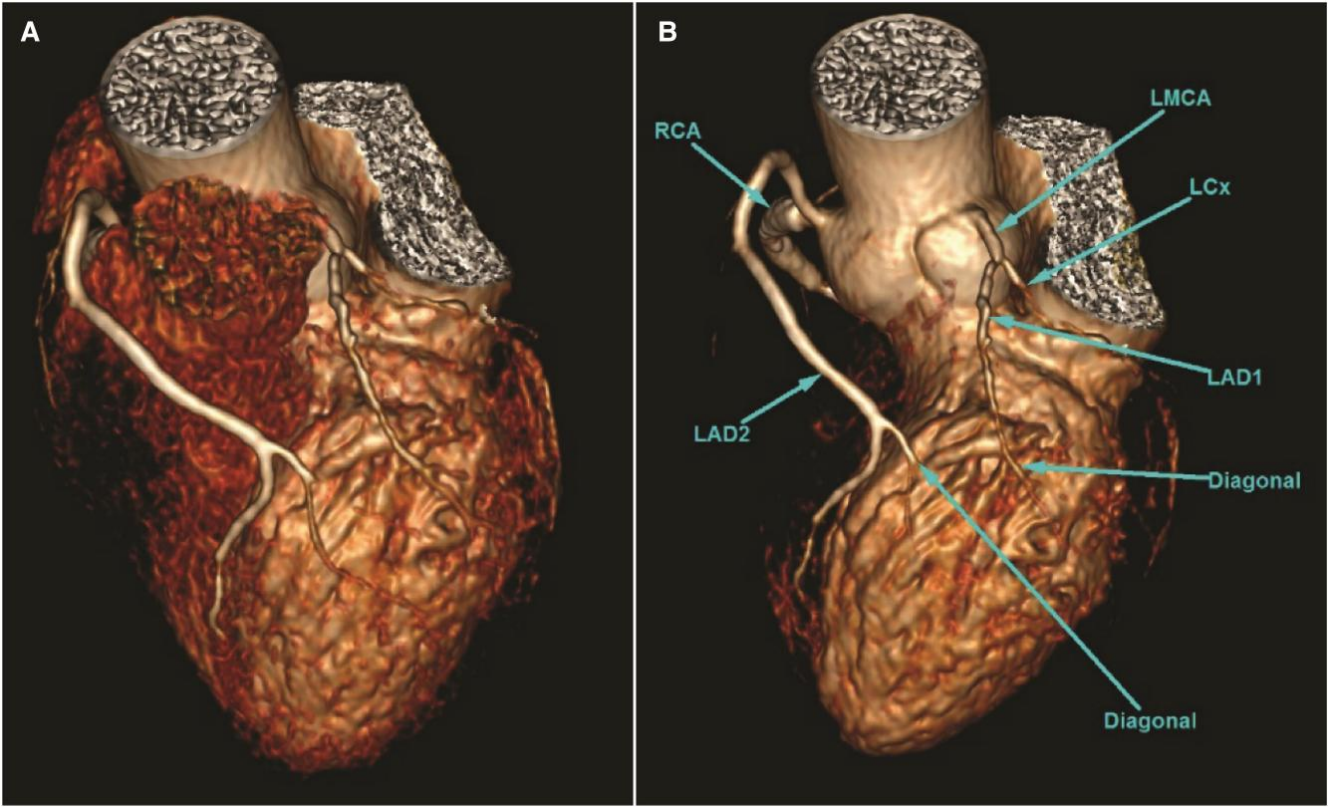
(*A*, *B*) Volume rendering computed tomography coronary image shows a type 4 dual left anterior descending artery. The short left anterior descending artery gives off the first major diagonal branch and ends in the proximal anterior interventricular groove. The aberrant long left anterior descending artery origin from the right coronary artery ostium is seen taking a pre-pulmonic course and enters the mid-anterior interventricular groove. LMCA: left main coronary artery; LAD1: short Left anterior descending artery; LAD2: Long left anterior descending artery; RCA: right coronary artery; LCX: left circumflex.

Subsequently, a pharmacoinvasive PCI was conducted after 24 h. The duration of the PCI procedure was 1 h, and a total of 150 mL of contrast was used during the procedure. The findings have revealed a normal LMCA, which bifurcates into a short LAD and left circumflex arteries. The short LAD gives rise to a diagonal branch before terminating early. Interestingly, the RCA exhibited an anomalous origin of LAD (long LAD) from the ostium of RCA without a significant lesion. Distal to the common ostium, there was a significant stenosis of RCA (90%–95%), proximal right posterior descending artery (RPDA) of 70%–80%, and proximal obtuse marginal 1 artery of the left circumflex artery (30%–50%) (*Figure 1*, [Supplementary material online, *Figure 2* will include all videos (2-1.avi, 2-2.avi, 2-3.avi, and 2-4.avi), documenting key procedural and clinical findings.]) Percutaneous coronary intervention (PCI) was performed on the RCA. Two drug-eluting stents were deployed in the RCA (4.0 × 15 mm) and RPDA (2.75 × 29 mm) with the thrombolysis in myocardial infarction flow score of 3.

After the procedure, the patient was well with no failure symptoms or chest pain. He was planned for dual antiplatelet therapy (oral aspirin and oral clopidogrel) for 12 months, followed by lifelong oral aspirin. Subsequently, the patient underwent computed tomographic coronary angiography (CTCA) to confirm the anomalous origin of LAD and other abnormalities of coronary arteries. Computed tomographic coronary angiography has showed that the LMCA has a normal origin and bifurcates into a short LAD and left circumflex artery. The short LAD gives rise to small septal perforators and terminates early by giving a branch to the diagonal artery. The long LAD originates from a common ostium with RCA from the right coronary sinus, which courses anterior to the root of the pulmonary artery before entering the anterior interventricular groove. The long LAD gives rise to the rest of the diagonal and perforator arteries (*Figure 2*). The patient has continued to attend the post-angiography follow-up visits for 1 year, remained in good health, and reported no chest pain, achieving a Canadian Cardiovascular Society score of zero. No follow-up echocardiography has been performed yet as the patient remains asymptomatic.

## Discussion

Primary percutaneous coronary intervention (PPCI) is the recommended reperfusion therapy for most patients with STEMI. However, timely access to PPCI is often hindered by geographic or logistical barriers. In such situations, a pharmacoinvasive approach—in which fibrinolysis is administered first, followed by immediate transfer to a PCI-capable hospital—can be an effective alternative. This strategy allows for a rescue PCI if fibrinolysis fails or a routine coronary angiography and PCI if the fibrinolysis is successful. In our case, thrombolysis was performed due to an anticipated delay in PCI availability, ensuring prompt reperfusion therapy. Patients with dual LAD vary from asymptomatic to experiencing chest pain, syncope, arrhythmia, and sudden cardiac death. The sudden cardiac death or acute coronary syndrome, such as myocardial infraction, is related to the course of the coronary artery that traverses between the aorta and pulmonary artery. It predisposes to compression when the great arteries dilate during intense strenuous activity. Coronary angiography is the gold standard investigation in diagnosing the anomaly. Anatomical anomaly of the coronary arteries may impose difficulty to interventional cardiologist to perform PCI. Awareness and recognition of dual LAD are important for several reasons. Inability to visualize the additional vessel (long LAD), when the long LAD originates from the right coronary sinus, could lead to misinterpretation of the variant anatomic features at routine coronary angiography of mid-LAD occlusion. Dual LAD identification is crucial for a number of reasons. When the long LAD originates from the right coronary sinus, it may be difficult to see additional vessel (long LAD) and could lead to misinterpretation of the short LAD as mid-LAD occlusion during routine coronary angiography. However, if there is no retrograde flow, one should look for a separate coronary artery flowing from the RCA—‘missing artery’. Furthermore, anomalous LAD emerging from the RCA may be misdiagnosed as a branch of the conus. However, the clue will be the presence of septal and diagonal branches. Searching for septal branches, good angiographic views with non-selective injections to left and right aortic cusps is helpful to identify coronary anatomy as well. Accurate knowledge of the anatomic features of the coronary arteries is essential for planning surgical vascularization. The anomaly is of great importance to surgeons while planning coronary artery bypass graft (CABG) surgery. Fortunately, in our case, the patient does not require CABG, as there was no disease in the left main or LAD coronary artery. However, before any intervention, a surgeon must have a thorough understanding of the coronary anatomy, especially regarding the origin and the course of an anomalous LAD and the variations of a dual LAD system. Inadequate revascularization, leading to post-operative angina and ischaemia, can result from a lack of this knowledge. If both the short and long LADs are significantly diseased, grafting both vessels is necessary to ensure proper revascularization of the heart’s anterolateral wall, interventricular septum, and apex. In addition, radiological imaging such as CTCA is beneficial to further define the course of an anomalous coronary artery, especially when the anomalous artery traverses between the great arteries or through the myocardium. It aids in clarifying temporal relationships between an anomalous coronary artery and surrounding structures as in this case.

In conclusion, type 4 dual LAD is an uncommon anomaly where the long LAD passes ahead of the major pulmonary artery root before entering the AIVS, which may have detrimental clinical consequences. Computed tomographic coronary angiography provides spatial information on the relationship between the long LAD and the neighbouring structures. Proper evaluation of the coronary anatomy is fundamental for successful PCI in this kind of coronary anomaly.

Our hospital is a tertiary care centre primarily serving the state and its surrounding areas, which together have a population of ∼1.8 million people. We have a dedicated cardiology unit staffed with two senior cardiologist with over 20 years of experience in cardiac interventions, performing ∼500 PCI procedures annually.

## Supplementary Material

ytaf032_Supplementary_Data

## Data Availability

The data underlying this article are available in the article and in its online Supplementary material.

## Lead author biography

Dr Ayman is currently a cardiology fellow at the Shendi University Hospital. He obtained his medical degree from Shendi University, followed by a residency in internal medicine at Universiti Sains Malaysia. He is now pursuing a fellowship in cardiology with a focus on interventional cardiology. During his residency, Dr Ayman gained extensive clinical experience in managing a wide range of cardiovascular diseases. He has been involved in several research. His research has been published in reputable cardiology journals.

## Notes

**Consent:** Written informed consent was obtained from the patient for publication of this case report and any accompanying images, in compliance with COPE guidelines.

**Funding:** None declared.
