# Supplementary material for: A rare case of dual left anterior descending artery type 4 in inferior myocardial infarction patient: a case report
Source: Eur Heart J Case Rep. 2025 Feb 5;9(2):ytaf032. doi: 10.1093/ehjcr/ytaf032 (PMC11830952; doi:10.1093/ehjcr/ytaf032)
Supplement: ytaf032_Supplementary_Data [file ytaf032_supplementary_data.zip › Serial ECGs.pdf]

On Arrival

ID: 278 For Use On EDAN/SPACELABS MS1R-32462

Male Years

Req. No. :

Department:

Azizi Ali
8842108.

29-10-2023 20:00:35

HR	: 60	bpm
P	: 68	ms
PR	: 212	ms
QRS	: 144	ms
QT/QTcBz	: 446/446	ms
P/QRS/T	: 7/77/95	°
RV5/SV1	: 0.748/0.404	mV

Diagnosis Information:

*** CONSIDER [illegible]UTE STEMI ***
Sinus rhythm with borderline 1st degree A-V block
Right bundle branch block
*** INFERIOR INFARCT - POSSIBLY ACUTE ***
Anterolateral ST elevation, CONSIDER ACUTE INFARCT
Low QRS voltages in precordial leads
Abnormal ECG

Ref-Phys. :

Report Confirmed by:

V1

DR. NUR ATHIRAH BT RAMLEE
(No. Pendaftaran Penuh MPM: 7144
Pegawai Perubatan Sarjana
Jabatan Perubatan Kecemasan
Hospital Universiti Sains Malaysia

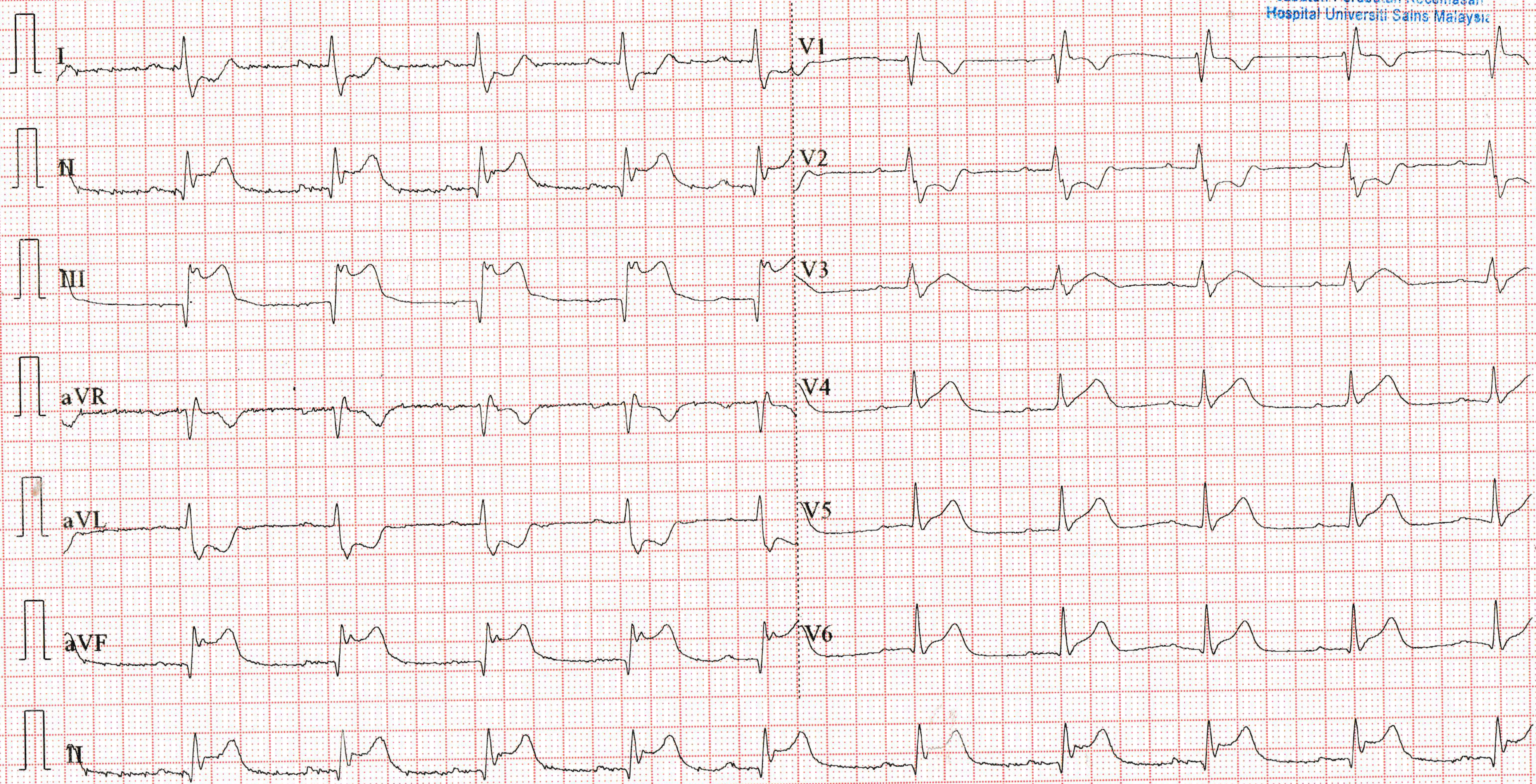

0.67~45Hz AC50 25mm/s 10mm/mV 2*5.0s+1r SE-1200Express V2.22 Glasgow V28.6.7

ID: 1209

For Use On EDAN/SPACELABS MS1R-32462 CE 0197 SONOMED

2023-10-29 20:11:32

Male Years
Req. No. :

Azizi Ali
B842108

HR	[illegible]64	bpm	
P	114	ms	
PR	: 192	ms	
QRS	: 142	ms	
QT/QTcBz	: 430/444		ms
P/QRS/T	: -10/67/95		°
RV5/SV1	: 0.350/0.000		mV

Diagnosis Informat
*** CONSIDER ACUTE STEMI ***
Sinus rhythm
Lead(s) unsuitable for analysis: V1 V2 V3
IV conduction defect
*** INFERIOR INFARCT - POSSIBLY ACUTE ***
Lateral ST-T abnormality suggests myocardial injury/ischemia
Abnormal ECG
Report Confirmed by:

DR NIMALLAH CHANDRAN
NO. PENDAFTARAN PENUH MPM : 67203
PEGAWAI PERUBATAN SARJANA
JABATAN KECEMASAN & TRAUMA
HOSPITAL UNIVERSITI SAINS MALAYSIA

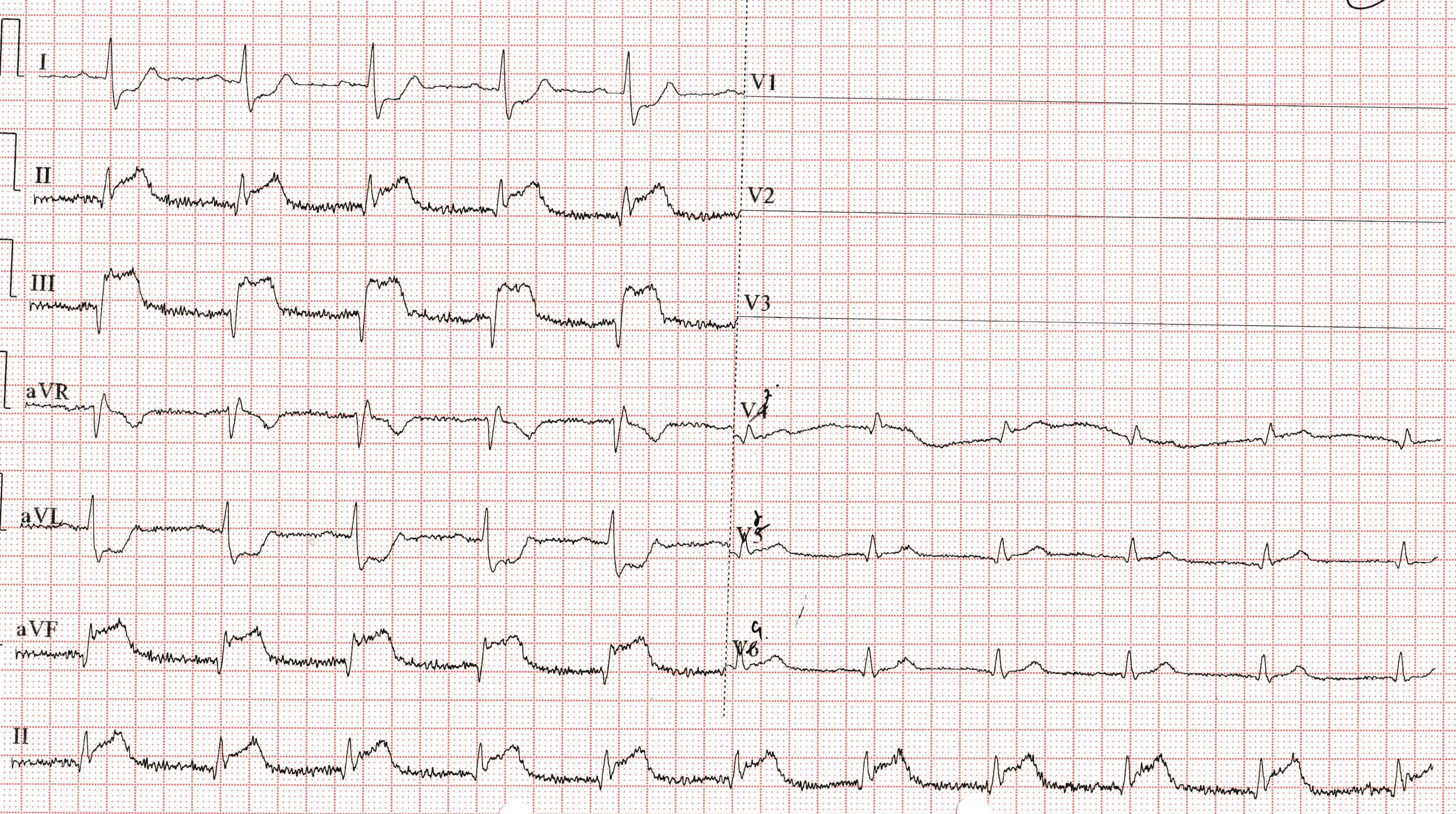

Posterior V7-V9

67~35Hz AC50 25mm/s 10mm/mV Lead Off 2*5.0s+1r SE-1200Express V2.22 Glasgow V28.6.7 JABATAN KECEMASAN I

ID: 1209 For Use On EDAN/SPACELABS MS1R-32462 2023-10-29 [illegible]0:07:21

Male Years

Req. No. :

Azizi ALI
B842108

HR	[illegible]59	bpm
P	[illegible]72	ms
PR	: 194	ms
QRS	: 140	ms
QT/QTcBz	: 436/432	ms
P/QRS/T	: -20/73/100	°
RV5/SV1	: 0.611/0.340	mV

Diagnosis Informati[illegible]
*** CONSIDER [illegible]TE STEMI ***
Sinus bradycardia
Possible sequence error: V1,V2 omitted
IV conduction defect
*** INFERIOR INFARCT - POSSIBLY ACUTE ***
*** LATERAL INFARCT - POSSIBLY ACUTE ***
Anteroseptal ST elevation, CONSIDER ACUTE INFARCT
Abnormal ECG
Report Confirmed by:

X2

DR NIMALLAH CHANDRAN
NO. PENDAFTARAN PENUH MPM: 67203
PEGAWAI PERUBATAN SARJANA
JABATAN KECEMASAN & TRAUMA
HOSPITAL UNIVERSITI SAINS MALAYSIA

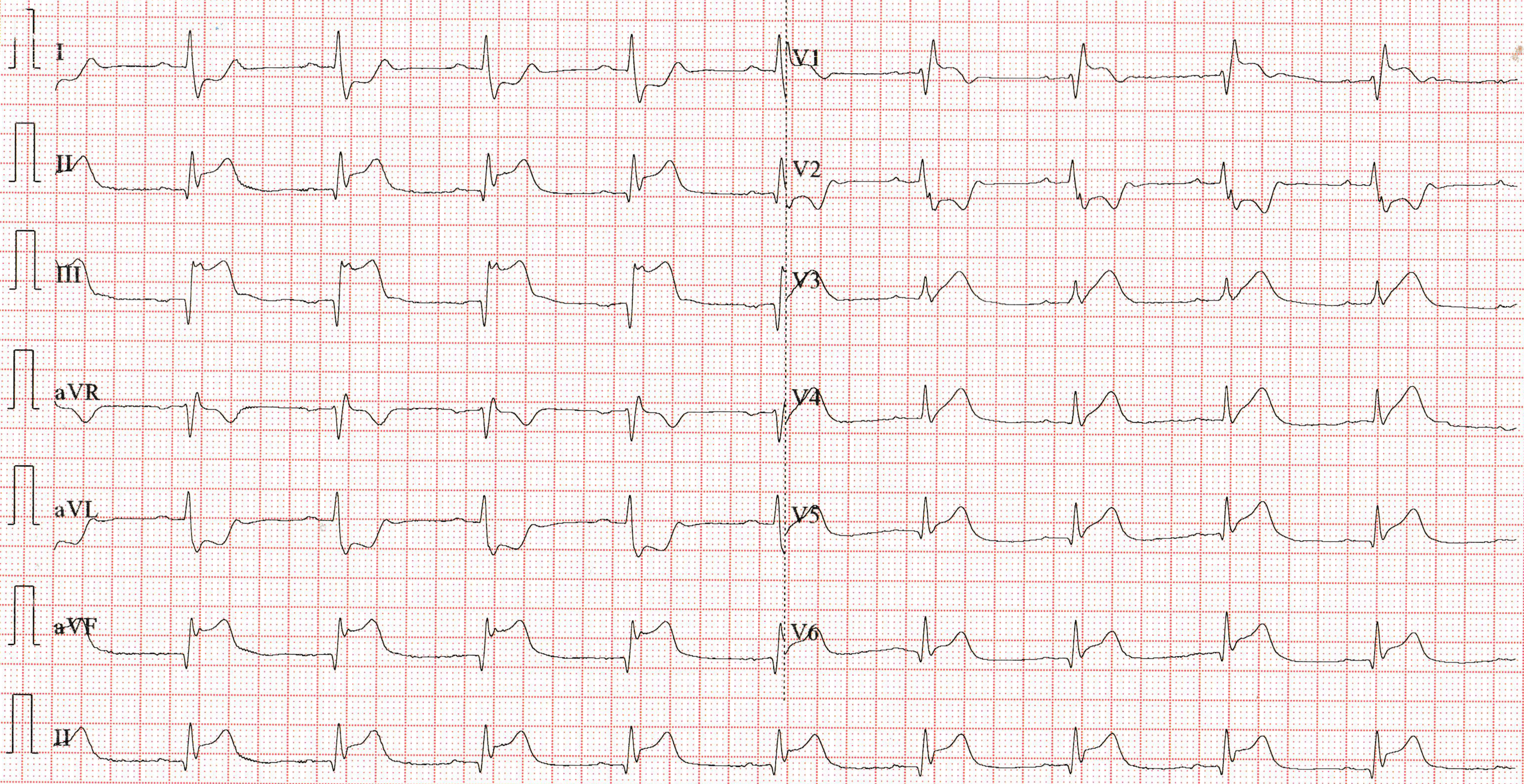

After 7 minutes

0.67~35Hz AC50 25mm/s 10mm/mV 2*5.0s+1r SE-1200Express V2.22 Glasgow V28.6.7 JABATAN KECEMASAN HUSM

For Use On EDAN/SPACELABS MS1R-32462 CE 0197 SONOMED

ID: 1209

Male Years

Req. No. :

Azizi Ali
B842108

2023-10-29 20:06:46

HR	: 78	bpm
P	: 102	ms
PR	: 192	ms
QRS	: 140	ms
QT/QTcBz	: 432/493	ms
P/QRS/T	: -45/72/100	°
RV5/SV1	: 0.624/0.339	mV

Diagnosis Information:

*** CONSIDER ACUTE STEMI ***

Possible ectopic atrial rhythm with interpolated PVC

Lead(s) unsuitable for analysis: V3

Right bundle branch block

*** INFERIOR INFARCT - POSSIBLY ACUTE ***

*** LATERAL INFARCT - POSSIBLY ACUTE ***

Anteroseptal ST elevation, CONSIDER ACUTE INF[illegible]

Low QRS voltages in precordial leads

Abnormal ECG

Report Confirmed by:

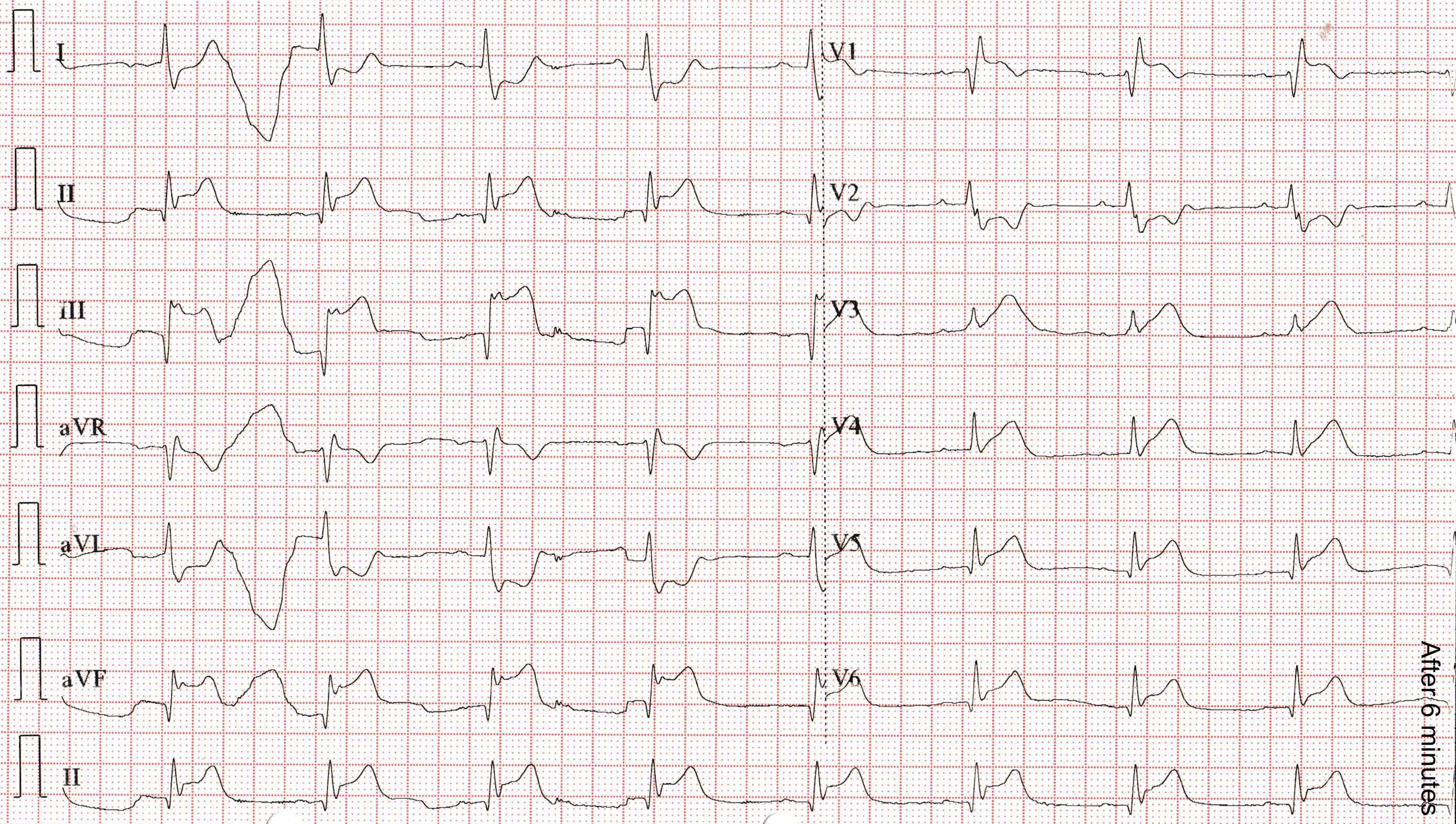

0.67~35Hz AC50 25mm/s 10mm/mV Lead Off 2*5.0s+1r SE-1200Express V2.22 Glasgow V28.6.7 JABATAN KE[illegible]

0 minute after thrombolysis

"0' post STK."

For Use On EDAN/SPACELABS MS1R-32462 CE 0197 SONOMED

ID: 1214 2023-10-29 21:14:42

Male Years

Req. No. :

A2121 ALI
B842108

HR	56	bpm
P	: 94	ms
PR	: 174	ms
QRS	: 164	ms
QT/QTcBz	: 478/462	ms
P/QRS/T	: 18/112/146	°
RV5/SV1	: 0.613/0.325	mV

Diagnosis Informati[illegible]
*** CONSIDER A[illegible]UTE STEMI ***
Sinus bradycardia with sinus arrhythmia with PAC(s)
Right axis deviation
Right bundle branch block
*** INFERIOR INFARCT - POSSIBLY ACUTE ***
Anteroseptal ST elevation, CONSIDER ACUTE INFARCT
Lateral ST-T abnormality may be due to myocardial ischemia
Abnormal ECG
Report Confirmed by:

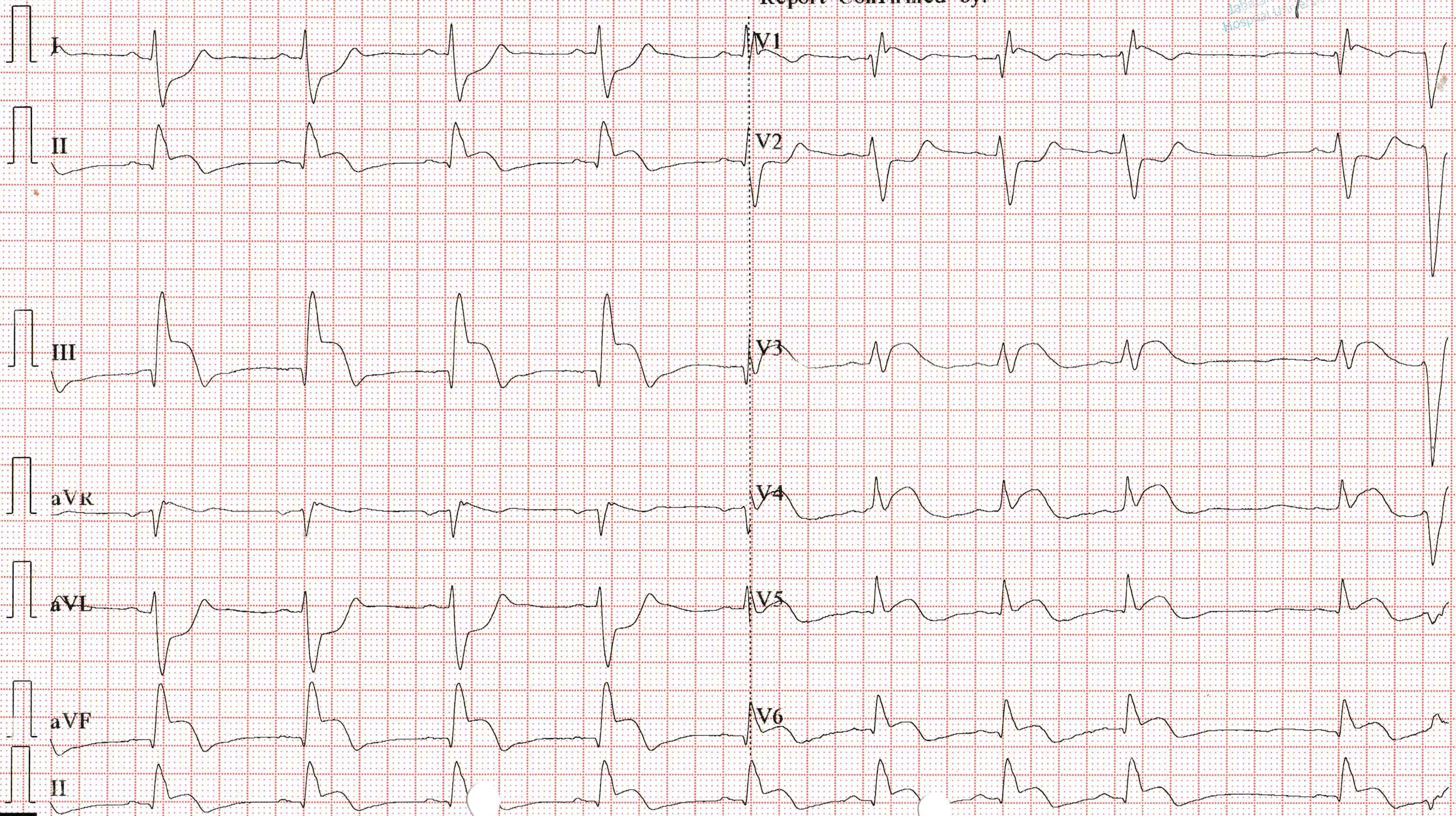

0.67-35Hz AC50 25mm/s 10mm/mV 2*5.0s+1r SE-1200Express V2.22 Glasgow V28.6.7 JABATAN KECEMASAN HUSM

30 minutes after thrombolysis

ID: 1215 For Use On EDAN/SPACELABS MS1R-32462 SONOMED 2023-10-29 21:44:05

Male Years A2121 ALI

Req. No. : B840/00

HR	:	58	bpm
P	:	124	ms
PR	:	180	ms
QRS	:	146	ms
QT/QTcBz	:	468/460	ms
P/QRS/T	:	21/97/122	°
RV5/SV1	:	0.564/0.332	mV

30 min

Diagnosis Informati[illegible]
*** CONSIDER [illegible]TE STEMI ***
Sinus bradycardia
Rightward axis
Right bundle branch block
*** INFERIOR INFARCT - POSSIBLY ACUTE ***
Lateral ST-T abnormality may be due to myocardial ischemia
Abnormal ECG
Report Confirmed by:

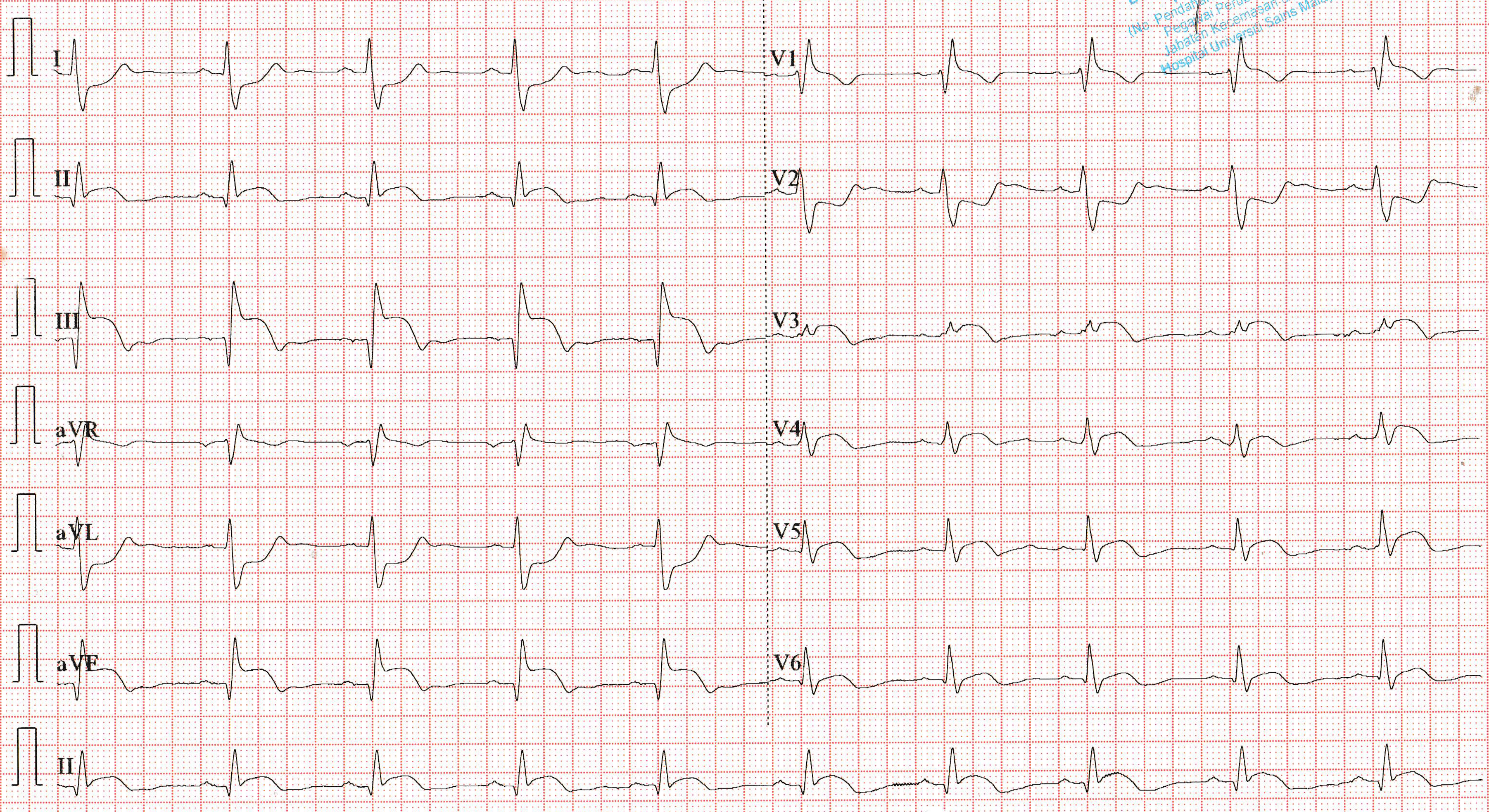

0.67-35Hz AC50 25mm/s 10mm/m[illegible] 2*5.0s+1r SE-1200Express V2.22 Glasgow V28.6.7 JABATAN KECEMASAN HUSM

219 KENZ R210 X 60Z-DM

Years

No. :

2023-10-29 22:47:43

HR	:	70	bpm
P	:	[illegible]	ms
PR	:	176	ms
QRS	:	94	ms
QT/QTcBz	:	404/436	ms
P/QRS/T	:	41/-6/84	°
RV5/SV1	:	0.684/0.426	mV

Diagnosis Information:

*** CONSIDER AC[illegible] STEMI ***

Sinus rhythm

*** INFERIOR INFARCT - POSSIBLY ACUTE ***

Anterolateral ST-T abnormality suggests myocardial injury/ischemia

Abnormal ECG

Report Confirmed by:

- 90min Post STK.

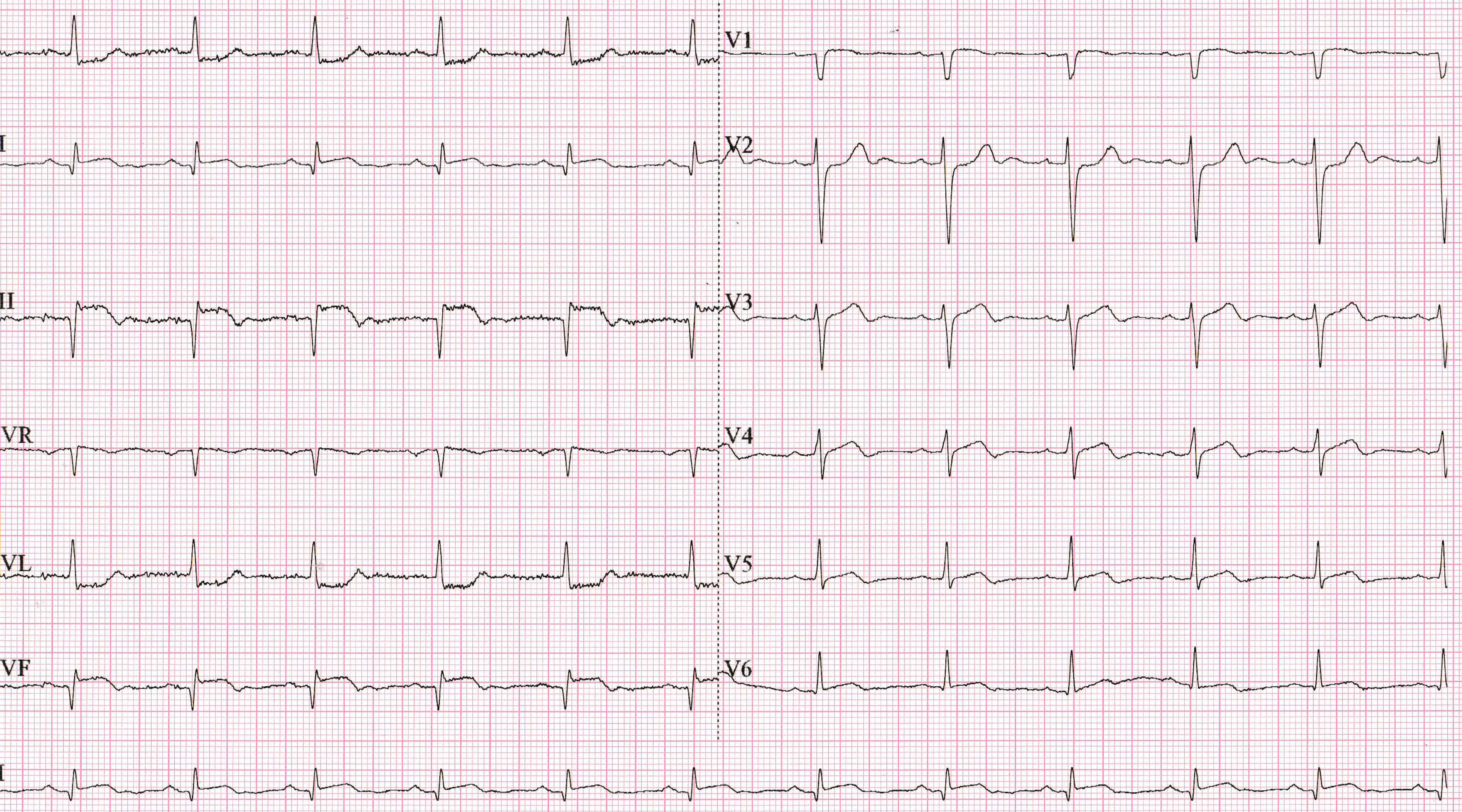

90 minute after thrombolysis

0.67~35Hz AC50 25mm/s 10mm/mV 2*5.0s+1r SE-1200Express V2.22 Glasgow V28.6.7 JABATAN KECEMASAN HUSM

SONOMED CE 0197

ID: 1217

Male Years
Req. No. :

[illegible]
B842108

2023-10-29 22:19:46

HR	:	62	bpm
P	:	102	ms
PR	:	176	ms
QRS	:	102	ms
QT/QTcBz	:	438/445	ms
P/QRS/T	:	17/-6/79	°
RV5/SV1	:	0.580/0.434	mV

Diagnosis In[illegible]mation:
*** CONSI[illegible] ACUTE STEMI ***
Sinus arrhythmia with PVC(s)
Possible sequence error: V2,V3 omitted
*** INFERIOR INFARCT - POSSIBLY ACUTE ***
Anterolateral ST-T abnormality may be due to myocardial ischemia
Abnormal ECG
Report Confirmed by:

60 min

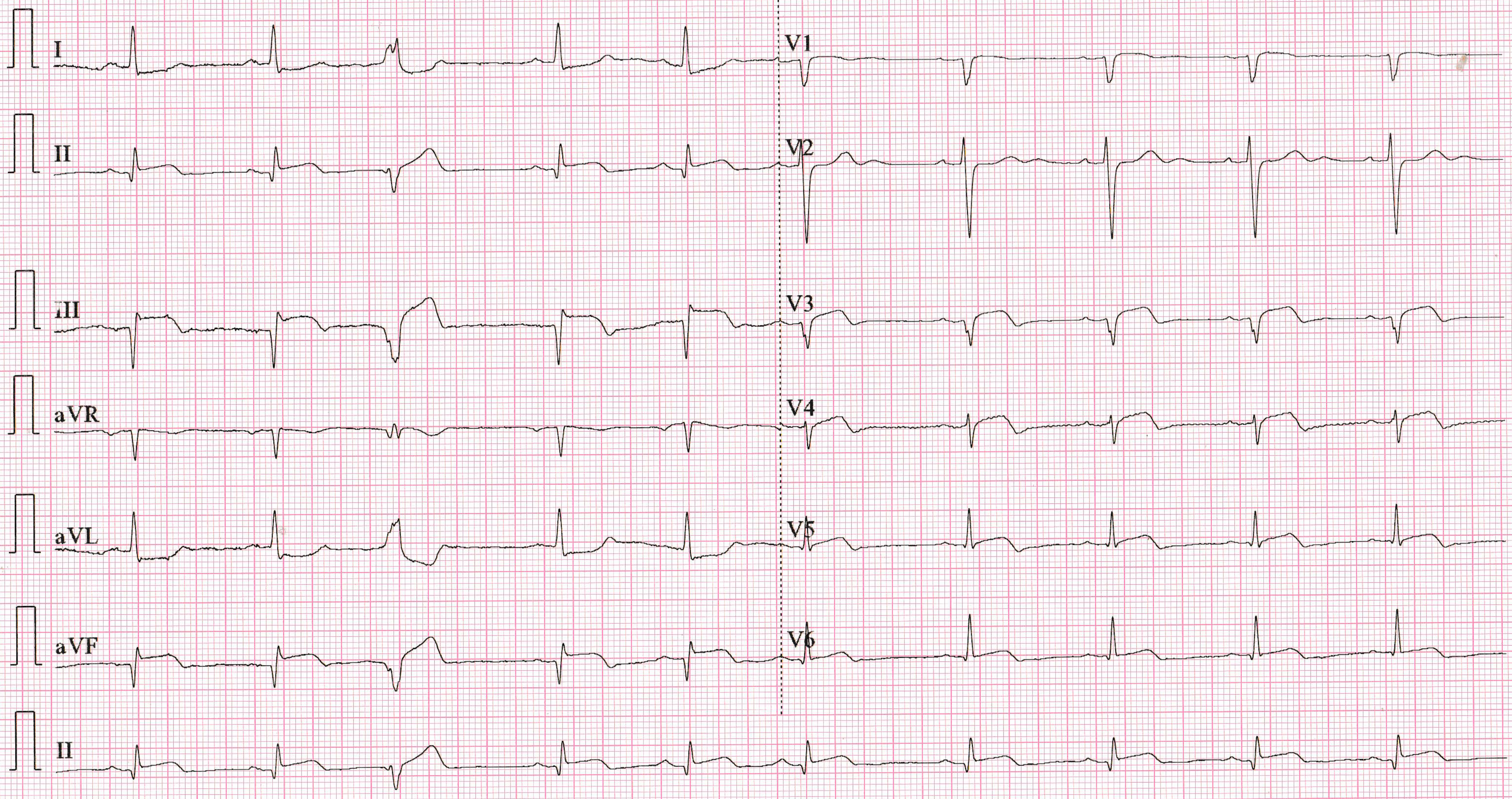

60 minutes after thrombolysis

0.67~35Hz AC50 25mm/s 10mm/mV 2*5.0s+1r SE-1200Express V2.22 Glasgow V28.6.7 JABATAN KECEMASAN HUSM

CE 0197 SONOMED
